# Case-Control Study of Fetal Microchimerism and Breast Cancer

**DOI:** 10.1371/journal.pone.0001706

**Published:** 2008-03-05

**Authors:** Vijayakrishna K. Gadi, Kathleen E. Malone, Katherine A. Guthrie, Peggy L. Porter, J. Lee Nelson

**Affiliations:** 1 Division of Clinical Research, Fred Hutchinson Cancer Research Center, Seattle, Washington, United States of America; 2 Division of Public Health Sciences, Fred Hutchinson Cancer Research Center, Seattle, Washington, United States of America; 3 Division of Basic Sciences, Fred Hutchinson Cancer Research Center, Seattle, Washington, United States of America; 4 Department of Medicine, University of Washington, Seattle, Washington, United States of America; 5 Department of Epidemiology, University of Washington, Seattle, Washington, United States of America; 6 Department of Biostatistics, University of Washington, Seattle, Washington, United States of America; Ordway Research Institute, United States of America

## Abstract

**Background:**

Prior pregnancy is known to protect against development of breast cancer. Recent studies have demonstrated that pregnancy has the capacity to establish small numbers of immunologically active fetal-derived cells in the mother, a phenomenon known as fetal microchimerism (FMc). We asked whether presence of FMc, routinely acquired during pregnancy, is a protective factor for breast cancer.

**Methodology/Principal Findings:**

DNA extracts from peripheral blood specimens were obtained from a population-based case-control study of risk factors for breast cancer in women 21 to 45 years old. Specimens were tested with quantitative PCR for presence and concentrations of male DNA presumed to derive from prior pregnancies with a male fetus. Odds ratios (OR) and 95% confidence intervals (CI) were estimated with consideration of multiple established reproductive and environmental risk factors for breast cancer. FMc results were generated on 99 parous women, 54 with primary invasive breast cancer and 45 general population controls. FMc prevalence was 56% (25/45) and 26% (14/54) in controls and cases, respectively. Women harboring FMc were less likely to have had breast cancer (OR = 0.29, 95% CI 0.11–0.83; p = 0.02, adjusting for age, number of children, birth of a son, history of miscarriage, and total DNA tested). In addition, FMc concentrations were higher in controls versus cases (p = 0.01). Median concentrations were 2 (0–78) and 0 (0–374) fetal genomes/10^6^ maternal genomes in controls and cases, respectively.

**Conclusions:**

Results suggest that the enigma of why some parous women are not afforded protection from breast cancer by pregnancy might in part be explained by differences in FMc. Mechanistic studies of FMc-derived protection against breast cancer are warranted.

## Introduction

Risk modifiers for development of breast cancer are both genetic and environmental. The known germline genetic risk modifiers identified thus far are few (e.g., BRCA1 and BRCA2) and overall account for only 2–10% of all cases [1]–[3]. Environmental risk modifiers including but not limited to reproductive factors, endogenous and exogenous hormones, anthropometric characteristics, and certain lifestyle factors are known to impact a woman's risk for breast cancer development. Mechanisms underlying these effects are still largely unclear and under active investigation. A prior history of pregnancy has been noted as a consistent protective factor in breast cancer but the magnitude of this reduced risk is relatively modest [4], [5]. Thus, it is conceivable that there are undefined characteristics of prior pregnancies that could underlie why some parous women experience a reduced risk of breast cancer while others do not.

We and others have shown that a natural consequence of normal pregnancy is the acquisition and stable persistence of small numbers of fetal cells in the mother's body, a phenomena known as fetal microchimerism (FMc) [6]–[9]. FMc has been identified in the peripheral blood within subsets of immune cells [7], [8]. Although the specific effects or consequences of FMc remain to be demonstrated in healthy women, FMc has been implicated in “alloimmunity” in some classically autoimmune diseases, for example systemic sclerosis and thyroiditis [8], [10]–[12]. One possible role of FMc in healthy women might be the provision of allogeneic immune surveillance for (pre-)malignant cells. Allogeneic cells, particularly mismatched or haploidentical cells, in the context of hematopoietic transplantation have been found to provide potent immune surveillance for both hematologic and solid tumor malignancies in a process referred to as graft-versus-tumor [13]–[15]. By integrating the aforementioned observations of FMc in autoimmunity and transplantation immunobiology, we reasoned that allogeneic FMc might provide immune surveillance for breast cancer in parous women. In a prospective, pilot study we previously identified a decreased prevalence of FMc in the peripheral blood mononuclear cells of parous women with breast cancer compared to controls [16]. We demonstrate here in a larger case-control study that women with breast cancer are deficient both by presence and quantity of FMc in the peripheral blood compared to carefully matched controls. This finding supports the hypothesis that parous women with breast cancer fail to harbor a potential source of naturally acquired allogeneic immunity.

## Methods

### Specimens

Specimens tested for FMc were obtained from women residing in the three county Puget Sound region of Western Washington State who previously participated in a population-based case-control study of breast cancer in women aged 21–44. The methods for this study have been described [17]. Briefly, cases (women with a first primary invasive breast cancer) were ascertained through the Cancer Surveillance System, a population based-cancer registry. Control participants (women without breast cancer) were identified by random-digit dialing in the same region and frequency matched for age. All participants completed an in-person structured interview detailing family, environmental, and reproductive histories and provided a peripheral blood specimen. Informed consent from participants of the parent study was documented with consent forms approved by the Fred Hutchinson Cancer Research Center Institutional Review Board. Samples from the larger case-control study included in this study were selected from women with non-metastatic disease, a history of at least one completed pregnancy, and no chemotherapy exposure before study phlebotomy.

For the current study, 100 participants were tested for FMc. One woman was excluded from final analysis because correlative clinical data for reproductive factors was not available. Altogether, 54 cases and 45 controls were evaluable for FMc.

### Procedures

FMc was identified and quantified in genomic DNA extracted from peripheral blood buffy coat specimens employing quantitative PCR for a Y chromosome specific gene, *DYS14*. Male DNA is assumed to derive from prior pregnancy with a male fetus. The approach permitted comparison of cases to controls while recognizing that the overall prevalence of FMc is underestimated as FMc from female fetuses could not be assessed. The method for testing male FMc using quantitative PCR has been previously described [7]. Testing for FMc was performed by a female technician (to minimize contamination from male DNA) blinded to the case-control status of the specimens. In brief, six aliquots of DNA from each specimen were tested for *DYS14*. Quantitative PCR reactions were performed on an ABI Prism 7000 running the manufacturer's software (SDS v1.2.3). Results from each aliquot were plotted by the software onto a simultaneously run calibration curve generated from known quantities of *DYS14*. An additional 2 aliquots from each specimen were tested for the beta-globin gene (present in two copies per cell) with results plotted similarly on a beta-globin calibration curve to provide an estimate of the total number of cells tested. After using a correction factor of 6.6pg of DNA per cell [18], data were expressed as a ratio of male (fetal) genome equivalents per 10^6^ host (maternal) genome equivalents. A minimum of 3.0×10^4^ genomes were tested in all 6 wells combined to insure sufficient material was examined for FMc. A maximum of 3.0×10^4^ genomes were tested in any single well because of PCR inhibition when greater input DNA is used. All wells read positive for *DYS14* sequence by the software were confirmed by individual amplification analysis in a blinded manner by one of the authors (VKG). For a specimen to be considered positive for FMc, a minimum of two wells were required to have surpassed the detection threshold for amplification.

### Statistical Analysis

The primary outcome for analysis was disease status (breast cancer case vs. control) and the principal predictor of interest was the presence of FMc. Factors examined as potential confounders or effect modifiers included age (at breast cancer diagnosis for cases, at time of study enrollment for controls), age at first birth, number of children, birth of a son, history of breastfeeding, miscarriage, abortion, oral contraceptive use, smoking status, age at menarche and number of cell equivalents tested for detection of FMc. A factor was defined as a confounder if there was a discrepancy of 10% or more in the estimated coefficient of interest between the multivariable model including the factor and the model without it. Differences in subject characteristics between groups were assessed via t-test for continuous variables and Chi-squared test for categorical factors. Logistic regression models were used to estimate the association between presence of FMc and disease status, while adjusting for age and other confounding factors.

FMc concentrations were further analyzed according to disease status. FMc concentrations were ranked and the ranks were used as the outcome for linear regression models to account for departures from normality in the distribution of the FMc values. Such transformation of the data preserves the ordering of values and gives estimates that are less sensitive to outlying values; however, model coefficients are no longer interpretable in terms of actual FMc values. The linear regression models were adjusted for age and other confounders.

Additional analyses were conducted to examine whether breast cancer-specific features were associated with FMc prevalence among the cases. Presence of FMc was treated as a binary outcome in logistic regression models, with age at diagnosis and various disease characteristics as the predictors. P-values from regression models were derived from the Wald test, and no adjustments were made for multiple comparisons. Analyses were performed on SAS software version 8 (SAS Institute, Inc., Cary, NC).

## Results

Baseline characteristics of breast cancer patients and control participants are shown in Table 1. The two groups did not differ with respect to most characteristics considered. Women diagnosed with breast cancer, however, were significantly older at the time they first gave birth (p = 0.03), an observation consistent with the original analysis of this case-control study [7]. Women with breast cancer were also more likely to have used oral contraception (p = 0.04) than control women.

**Table 1:** Baseline characteristics of breast cancer cases and controls

	Cases (54)	Controls (45)
	N (%)	N (%)
**Race**		
Caucasian/Hispanic	48 (89)	41 (91)
Other	6 (11)	4 (9)
**Median age in years (range)**	41 (35–44)	41 (31–44)
**Smoking status**		
Never	30 (56)	23 (51)
Former	8 (15)	9 (20)
Current	16 (30)	11 (24)
Unknown	0	2 (4)
**Age at first birth (years)** *		
<20	9 (17)	12 (27)
20–29	31 (57)	29 (64)
≥30	14 (26)	4 (9)
**Number of children**		
1	13 (24)	12 (27)
2	24 (44)	18 (40)
3 or more	17 (32)	15 (33)
**Gave birth to a son**	42 (78)	32 (71)
**At least one pregnancy loss** †	29 (54)	21 (47)
**Number of miscarriages**		
0	35 (65)	33 (73)
1	12 (22)	8 (18)
2 or more	7 (13)	4 (9)
**Number of abortions**		
0	40 (74)	32 (71)
1	10 (19)	8 (18)
2 or more	4 (7)	5 (11)
**Breastfed at least 2 weeks**		
No	13 (24)	14 (31)
Yes	41 (76)	31 (69)
**Age at menarche (years)**		
8–12	27 (50)	18 (40)
>13	27 (50)	27 (60)
**Duration oral contraception use (years)** ‡		
<1	6 (11)	14 (31)
1–4	24 (44)	14 (31)
≥5	24 (44)	17 (38)

* Age at first birth older in cases than in controls (p = 0.03);

† Pregnancy loss includes miscarriage, abortion, tubal pregnancy and stillbirth;

‡ Exposure to oral contraception greater in cases than in controls (p = 0.04).

Overall, FMc was found in 39 women, including 25 of 45 controls (56%) and 14 of 54 breast cancer patients (26%). Table 2 shows the prevalence of FMc and the association of FMc and risk of breast cancer. Compared to women who were FMc-negative, women harboring FMc were significantly less likely to have had breast cancer (OR = 0.20, 95% CI 0.08–0.53; p = 0.001, adjusting for age, number of children, birth of a son and history of miscarriage). The total number of cell equivalents tested for detection of FMc was higher in the controls than in the breast cancer cases (mean 8.2×104 (95% CI 7.2–9.2×10^4^) in controls versus 5.6×104 (95% CI 4.9–6.3×10^4^) in cases). This factor acted as a moderate confounder, slightly diminishing the effect of FMc in the final model (OR = 0.29, 95% CI 0.11–0.83; p = 0.02).

**Table 2:** Risk of breast cancer and FMc

Presence of FMc	Proportions by Disease Status		OR (95% CI)	p
	No. Cases (%)	No. Controls(%)		
No	40 (74)	20 (54)	1.0	–
Yes	14 (26)	25 (56)	0.20(0.08–0.53)	0.001*
			0.29 (0.11–0.83)	0.02**

* Adjusted for age, number of children, birth of a son, history of miscarriage;

** adjusted for above and total number of cell equivalents tested.

**Figure 1:**
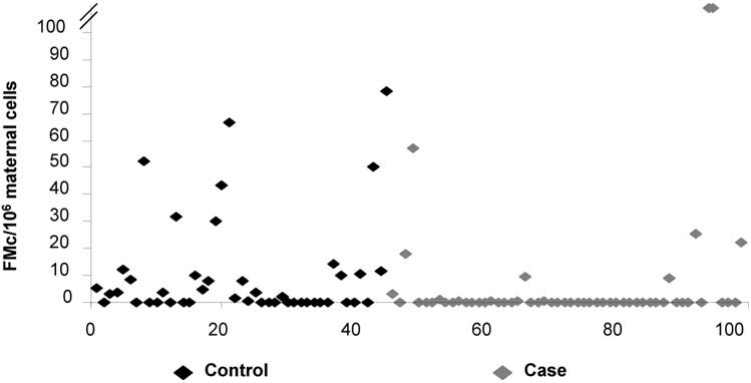
FMc concentrations in peripheral blood buffy coat (fetal genomes per 10^6^ maternal genomes). Median FMc concentrations were 2 (0–78) and 0 (0–375) in controls and cases, respectively. Two outlying values among the cases (277 and 374) are shown truncated.

Concentrations of FMc in peripheral blood in women with and without breast cancer are depicted in Figure 1. Median concentrations were 2.0 versus 0 FMc cells per million maternal cells in healthy women and breast cancer patients, respectively. FMc concentrations were significantly higher in the controls than in the women with history of breast cancer (p = 0.01) in a model of the ranked values adjusted for age, number of children, birth of a son, history of miscarriage, oral contraceptive use, and total number of genomes tested.

Among cases, analyses were conducted to examine whether breast cancer specific features were associated with FMc prevalence (Table 3). The results suggest that women without FMc were more likely to have advanced disease features than women with FMc although none of these associations achieved statistical significance.

**Table 3:** Prevalence and odds of harboring FMc according to disease characteristics

	FMc (14)	No FMc (40)	Adjusted*
	N (%)	N (%)	OR (95% CI)
**AJCC** † ** stage**			
I	11 (33)	22 (67)	1.0
IIA or higher	3 (14)	18 (86)	3.1 (0.7–14.3)
**Tumor size (cm)**			
≤2	12 (30)	28 (70)	1.0
2–5	2 (17)	10 (83)	1.7 (0.3–9.7)‡
>5	0	2 (100)	–
**Nodal status**			
Negative	11 (29)	27 (71)	1.0
Positive	3 (19)	13 (81)	2.1 (0.4–9.8)
**Estrogen receptor**			
Positive	13 (29)	32 (71)	1.0
Negative	1 (11)	8 (89)	6.6 (0.6–73.5)
**Progesterone receptor**			
Positive	14 (31)	31 (69)	–§
Negative	0	9 (100)	–§
**Average ratio of Ki-67**			
0–24%	13 (30)	30 (70)	1.0
25–100%	1 (9)	10 (91)	3.1 (0.3–28.3)
**C-erbB-2**			
Negative	7 (26)	20 (74)	1.0
Positive	7 (26)	20 (74)	1.5 (0.4–5.7)
**Histology**			
Ductal	12 (27)	32 (73)	1.0
Other	2 (20)	8 (80)	1.1 (0.2–6.6)
**Histology grade**			
Low	6 (38)	10 (62)	1.0
Intermediate or High	8 (22)	28 (78)	2.1 (0.5–8.3)

* Adjusted for age at diagnosis;

† AJCC = American Joint Committee on Cancer;

‡ Tumor sizes of 2–5 and >5 cm combined for comparison to ≤2 cm;

§ Odds ratio cannot be calculated.

## Discussion

In this case-control study of breast cancer among parous women, women with breast cancer were more frequently deficient in FMc when compared to control women. Both overall prevalence and quantity of FMc were significantly different between parous women with and without breast cancer. Other known risk modifiers for breast cancer such as age at first pregnancy, history of oral contraceptive use, breast feeding, and smoking did not meaningfully confound the overall association of FMc absence with breast cancer. Furthermore, in case-only analyses, women with adverse breast cancer features (for example, higher stage or hormone receptor negativity) consistently had lower prevalence of FMc although no single characteristic reached statistical significance because power was limited for such analyses.

In prior pilot studies we similarly found a decreased prevalence of FMc in women with breast cancer [16]. When comparing the current data to our prior pilot study, the current data strengthens the overall inverse association of FMc and breast cancer. First, the study here considers more cases for analysis and permitted both examination of proportions and quantities of FMc when compared to the prior pilot study. The current findings replicate the principal finding of the pilot and add to it by demonstrating a difference in FMc concentrations between cases and controls. Second, because we utilized specimens from a matched case-control study, reproductive and other risk factors were more similar than in the prior pilot and allowed for multivariable modeling to adjust for any potential confounders.

When comparing the current data to prior studies of FMc, some additional differences merit discussion. FMc results could vary depending on the compartment of blood tested. Most prior studies of FMc examined Ficoll-purified peripheral blood mononuclear cells. To our knowledge, there are no studies of FMc in buffy coat. The overall prevalence and concentrations of FMc in controls described here were somewhat higher when compared to findings from Ficoll-purified cells. The key constituents more greatly represented in buffy coat compared to Ficoll-purified cells are large granular cells such as neutrophils or dendritic cells. Our data then indirectly suggest that FMc in healthy women might be represented in cells that are part of the innate immune response and/or in cells involved in antigen presentation. Second, in prior work, we have been able to employ an extensive panel of allelic quantitative PCR assays to catalog all potential sources of FMc regardless of sex of the child [19]. We did not have access to samples from children of probands in the current study for the purpose of polymorphism typing to identify informative assays for all potential FMc. On the other hand, control women and women with breast cancer were similar for having given birth to children of either sex and for total number of children.

The protection from breast cancer afforded by parity is incomplete. We provide data that suggest there is a failure to acquire or maintain allogeneic immune cells (FMc) transmitted from the fetus to the mother during pregnancy in women who develop breast cancer. These allogeneic cells have the potential to participate in immune surveillance for breast or other cancers as observed in clinical hematopoietic stem cell transplantation. Additional studies are required to identify the specific mechanisms by which FMc could participate in the protection from breast or other cancers. Knowledge gained from mechanistic studies may contribute to the design of allogeneic vaccine-based or adoptive immunotherapy for breast cancer. We acknowledge that replication of our findings in larger study populations is required because the sample size is small. Nonetheless, if our results are replicated, testing for FMc could be used to identify healthy women who might be at risk for developing breast cancer. Finally, the work presented here is of potential interest to evolutionary biologists as it provides additional support in human females for the concept of long-term fitness benefit through reproduction.
